# Overexpression of Transmembrane Phosphatase with Tensin homology (TPTE) in prostate cancer is clinically significant, suggesting its potential as a valuable biomarker

**DOI:** 10.1007/s00432-024-05694-6

**Published:** 2024-03-28

**Authors:** Nahid Zainodini, Maryam Abolhasani, Monireh Mohsenzadegan, Mohammad M. Farajollahi, Elham Rismani

**Affiliations:** 1 Department of Medical Biotechnology, School of Allied Medical Sciences, Iran University of Medical Sciences (IUMS), Hemmat Highway, Tehran, Iran; 2 Department of Pathology, School of Medicine, Iran University of Medical Sciences, Tehran, Iran; 3 Hasheminejad Kidney Center, Iran University of Medical Sciences, Tehran, Iran; 4 Department of Medical Laboratory Sciences, Faculty of Allied Medical Sciences, Iran University of Medical Sciences (IUMS), Hemmat Highway, Tehran, Iran; 5 Molecular Medicine Department, Biotechnology Research Center, Pasteur Institute of Iran, Tehran, Iran

**Keywords:** TPTE protein, Prostate cancer (PCa), Immunohistochemistry, Benign prostatic hyperplasia (BPH), Targeted therapy

## Abstract

**Purpose:**

Cancer testis antigens (CTAs) are a family of proteins typically expressed in male testicles but overexpressed in various cancer cell types. Transmembrane Phosphatase with Tensin homology (TPTE) is expressed only in the testis of healthy individuals and is a member of the family of CTAs. The current study, for the first time, examined the significance of TPTE expression in prostate cancer (PCa) tissues by generating a novel antibody marker targeting TPTE protein.

**Methods:**

Polyclonal antibodies were prepared for TPTE-p1 and TPTE-p2 peptides, which are derived from the extracellular domains of TPTE. Anti-TPTE-p2 antibody was then used to study the extent and pattern of TPTE expression in 102 PCa and 48 benign prostatic hyperplasia (BPH) tissue samples by immunohistochemistry. The viability of cancer cell lines (PC-3 and MCF-7 cells) was also evaluated in the presence of anti-TPTE-p2 antibody using the MTT test.

**Results:**

The immunohistochemical analysis demonstrated a significant increase in cytoplasmic and membrane TPTE expression in the PCa samples compared to the BPH group (both P < 0.0001). Cytoplasmic TPTE expression was positively correlated with Gleason score and PSA levels (P = 0.03 and P = 0.001, respectively). Significant correlations were identified between the levels of PSA and perineural invasion and the membrane expression (P = 0.01, P = 0.04, respectively). Moreover, anti-TPTE-p2 antibody inhibited PC-3 and MCF-7 cells proliferation compared to the control group for 24 h (P < 0.001 and P = 0.001, respectively) as well as for 48 h (*P* = 0.001 and *P* = 0.001, respectively).

**Conclusion:**

Our findings indicate that increased TPTE expression is associated with progression of disease. The ability of anti-TPTE-p2 antibody to recognize and target the TPTE protein makes it a potential biomarker to assess and/or target the PCa.

## Introduction

Prostate cancer (PCa), the second most frequent cancer among men, is a heterogeneous disease and the fifth cause of death in men (D’Elia et al. 2022; Torre et al. 2016). Approximately 1,414,259 people were diagnosed with PCa in 2020, and 375,304 of them died. This number will almost double to 2.3 million cases and 740,000 deaths in 2040 worldwide (Chung et al. 2019; Culp et al. 2020; Ferlay et al. 2019; Sung et al. 2021). While prostate-specific antigen (PSA)-based screening for PCa has had a beneficial effect in reducing PCa mortality, it has also resulted in the problem of overdiagnosis, overtreatment, and a substantial number of unnecessary biopsies (Srivastava et al. 2019). More precise markers can identify individuals with aggressive forms of the disease, enabling tailored treatment approaches. Novel markers may also reveal new therapeutic targets, leading to targeted treatments that disrupt cancer cell pathways or enhance immune response (Nandana and Chung 2014). Overall, the search for new markers aims to enhance diagnostic accuracy, refine prognostic assessments, and develop more effective and personalized therapeutic strategies. Tumor markers, which are substances produced in excessive amounts by the body or cancer cells as cancer develops and progresses, offer valuable information for the diagnosis, prognosis, and prediction of different cancers (Saoud et al. 2020; Yang et al. 2023).

CTAs have emerged as a large family of tumor‐associated antigens expressed in human tumors of different histological origin, that warrant further investigation, as they have not been thoroughly explored (Kulkarni et al. 2012; Wei et al. 2020). They are a family of proteins by their importance in development and in cancer immunotherapy and typically expressed in male testicles, though they involve in many other cancer cell types (Yang et al. 2015). Based on the available information in the CT database and GeneBank, more than 200 CTAs are found which are classified into 40 groups (Chong et al. 2022; Nin and Deng 2023). Because of their tumor-restricted expression, low level of MHC class I, and low immunogenicity, CTAs are considered as ideal targets for tumor specific immunotherapeutic approaches and prompted the development of several clinical trials of CTAs-based vaccine therapy (Salmaninejad et al. 2016). Previous studies have shown its expression in various malignancies is heterogeneous and often correlates with progression of tumor, invasion, and metastasis (Gjerstorff et al. 2016; Shang et al. 2014).

TPTE is a gene belonging to members of the CTA family expressed only in the testis of healthy individuals but absent in healthy tissues (Atanackovic et al. 2006). It encodes 551 amino acids with an approximate weight of 64 kDa and possesses a minimum of two transmembrane domains, a phosphatase domain, and a domain homologous to tensin (Atanackovic et al. 2006; Dong et al. 2003). It exhibits a genetic sequence resemblance to the extensively expressed suppressor, phosphatase and tensin homolog (PTEN). Notably, TPTE distinguishes itself through an N-terminal extension featuring three transmembrane regions, leading to its alternate designation as PTEN2 (Leslie et al. 2007). The human genome contain many copies of TPTE located on acrocentric chromosomes 13, 15, 21, and 22 and the Y chromosome; and, only one copy on chromosome 21 can be expressed (Chen et al. 1999). The function of TPTE remains incompletely elucidated, yet it is presumed to contribute to signal transmission through tensin and interaction with the cytoskeleton (Singh et al. 2008). The presence of TPTE protein has been detected in diverse cancer types, such as hepatocellular carcinoma (Dong et al. 2003), lung cancer (Kuemmel et al. 2015), and ovarian cancer (Adepiti and Odunsi 2022). Earlier investigations have provided evidence that TPTE exhibits elevated expression in androgen-independent PCa cell lines, as opposed to hormone-sensitive cells. Differences in TPTE protein expression show promise as a valuable tool for diagnosing and predicting both localized and metastasized cancer (Singh et al. 2008). Previous research has highlighted the presence of TPTE on the cellular membrane (Walker et al. 2001), designating it as a viable candidate for targeted therapy in treating PCa. This study represents the inaugural investigation assessing TPTE expression patterns in a series of PCa samples, employing newly generated anti-TPTE antibodies for immunohistochemistry (IHC) analysis. Examining the association between TPTE expression and various clinicopathological features in patients with PCa, our study aimed to identify a potential protein marker for the assessment and management of the disease.

## Material and methods

### Bioinformatics analysis

To generate antibodies against the TPTE protein, a sequence corresponding to the TPTE was obtained from the Uniport database (ID: P56180). The extracellular regions of the protein sequence were subjected to computational modeling using the Swiss-model program, which employs homology-based modeling techniques to generate a three-dimensional structure. The IEDB website algorithms were employed to assess both linear and non-linear epitopes linked to B-cells, aligning with the extracellular regions of the protein sequence. Peptide fragments, spanning 15–20 amino acids, were extracted, and subjected to analysis for physicochemical properties, antigenicity, and allergenicity. Ultimately, two sequences TPTE-p1 (N-IYSIPRYVRDLKIQIEMEK-C) andTPTE-p2 (N-ELDNLHKQKARRIYPSDF-C) were selected and synthesized (Pepmic CO, China) for this study.

### Polyclonal Ab production

The peptide sequences of TPTE were linked to bovine serum albumin (BSA) from Sigma-Aldrich, USA, to enhance immunogenicity. To fulfill this purpose, a sulfo-SMCC (Sulfo-succinimidyl-4-(N-Maleimidomethyl) Cyclohexane-1-Carboxylate) linker from Sigma-Aldrich, USA, was utilized. In summary, sulfo-SMCC was dissolved in distilled water and combined with BSA in a pH 7.2 phosphate buffer solution. The resulting mixture underwent incubation at room temperature (RT) for one hour. Subsequently, the product obtained underwent purification through a desalting column filled with Superdex 200 Prep Grade from Sigma-Aldrich, USA. The maleimide activated protein was then combined with peptide solutions with a molar ratio of 1:30 (BSA to peptide), in a phosphate buffer solution (pH 7.2). After incubating at RT for two hours, the reaction underwent dialysis at 4°C in phosphate-buffered saline (PBS) for 12 h. Subsequently, the analysis of the conjugated products was conducted using sodium dodecyl sulfate–polyacrylamide gel electrophoresis (SDS-PAGE) (Mohsenzadegan et al. 2013). The modified proteins were subsequently used to immunize animals to produce antibodies. To produce polyclonal antibodies, four adult female New Zealand white rabbits, sourced from the Pasteur Institute in Iran, were utilized. The conjugated protein was thoroughly dissolved in complete Freund’s adjuvant from Sigma-Aldrich, USA. This solution was then injected into various areas of the rabbits’ backs during the immunization stage. Booster injections, consisting of a conjugated peptide solution in Freund’s incomplete adjuvant, were administered one month after the initial injection to attain the desired antibody titer. Blood samples were collected before the first injection and 10 days after each booster injection, from which serum samples were prepared (Mohsenzadegan et al. 2015).

### Immunoreactivity analysis of the rabbit sera by ELISA

The ELISA technique was employed to assess the immunoreactivity of rabbit sera. Antibody concentrations were measured through an indirect ELISA method. Initially, 96-well flat-bottom microtiter plates were loaded with TPTE-p1 and p2 peptides (1μg/well) and incubated for one hour at 37 °C to quantify antibody levels. The wells were blocked using 5% skim milk in PBS (pH 7.4) and incubated for 1 h at RT. Plates were washed three times with wash solution (PBS plus 5% Tween 20), incubated with 100 μl of immunized rabbit serum at serial dilutions (1/400, 1/800, 1/1600, 1/3200, 1/6400, and 1/12800), and then incubated for 30 min at 37 °C. Subsequently, we introduced a secondary antibody, goat anti-rabbit IgG, conjugated with HRP from Sigma-Aldrich, USA, at a 1:10,000 dilution. This was followed by a 30-min incubation at 37 °C. After each step, the plates underwent a washing process with a wash solution. Subsequently, 100 μl of the 3,3′,5,5′-tetramethylbenzidine (TMB) substrate reagent from Dako, Denmark, was added to all wells. The color development was halted by the addition of 0.2 M H_2_SO_4_ (100 μl). The outcomes were analyzed by ELISA reader using optical density of 450 nm (Dana Tajhiz, Iran). TPTE-p1 and p2 immobilized on NHS-Activated Agarose beads (Pierce Chemicals Co., USA) and specific anti-peptide antibodies were purified from rabbit sera using the suggested protocol (Mohsenzadegan et al. 2015).

### Sample preparation

A total of 150 paraffin-embedded tissue samples, comprising both PCa and BPH as the control group, were procured from patients at Hasheminejad Urology-Nephrology Hospital in Iran, spanning the years 2016 to 2017. The sample set encompassed 102 PCa tissues and 48 BPH tissues. Notably, none of the patients underwent preoperative treatments, such as hormonal therapy or radiotherapy. The PCa samples were categorized based on the Gleason grading system. Comprehensive analysis involved the examination of medical records and slides stained with hematoxylin and eosin (H&E) to extract details on specific pathological and clinical features, including patient age, serum PSA levels, and Gleason score. Survival data for patients, including disease-specific survival (DSS), overall survival (OS), and biochemical recurrence (BCR), were meticulously documented until the end of 2022. Whereas OS is characterized as the duration from the surgical procedure to the individual's demise (Rygalski et al. 2021). DSS is delineated as the timeframe commencing with the surgical intervention and extending until either the patient's cancer-induced death or the most recent follow-up (Shoup et al. 2003). And BCR is defined as the period from the primary treatment or radical prostatectomy to the point when serum PSA levels exhibit an increase beyond 0.2 ng/ml (Matsumoto et al. 2018).

### Immunohistochemical analysis

To examine TPTE expression in tissue samples, immunohistochemical staining was conducted on paraffin-embedded sections of PCa. After deparaffinization and rehydration of the tissues, endogenous peroxidase activity was quenched by immersing the tissue in methanol containing 0.3% hydrogen peroxide for 15 min. Heat-induced epitope retrieval was performed using a pressure cooker and a solution comprising 10 mM sodium citrate and 0.05% Tween 20 at pH 6.0. Following cooling at RT, the sections underwent two PBS washes. Incubation with the primary antibody (anti-TPTE-p2 Ab) occurred at serial dilutions of 1:20, 1:40, and 1:80 in a humid chamber at 4 °C, with the optimal dilution determined as 1:20. Subsequently, incubation with poly-HRP-goat anti-mouse/rabbit IgG (DPVB110HRP, BrightVision, Netherlands) was carried out at RT for 30 min. The samples were visualized using 3,3ˊ-diaminobenzidine (DAB, Dako, Denmark), counterstained with hematoxylin (Dako, Denmark), and observed under an optical microscope (Nikon, Japan) (Shafiei et al. 2019). Testis tissue served as a positive control for validating the anti-TPTE-p2 antibody, while the absence of the primary antibody in PCa tissue acted as the negative control. To validate the antibody’s specificity in immunohistochemical staining, the TPTE-p2 peptide underwent a pre-incubation of 2 h with the anti-TPTE-p2 antibody at RT before application to the sample. The assay utilized peptide concentrations ranging from 1 to 20 μg/ml, combined with the TPTE-p2 antibody at an optimal dilution of 1:20.

### Scoring system

An uninformed professional pathologist assessed the immunohistochemical staining of tissue slides through a semi-quantitative scoring system. Staining intensity was categorized as negative, weak, moderate, or strong. The percentage of TPTE-positive cells received grades: 0 (no staining), 1 (< 50% positive cells), 2 (50–80% positive cells), and 3 (> 80% positive cells). The total score was determined using the histochemical score (H-score) for each case, calculated by multiplying the staining intensity by the percentage of positive cells, resulting in an ultimate score ranging from 0 to 300. The mean H-scores, namely 188 for cytoplasmic, 134.88 for membrane, and 191.67 for nuclear expression, were employed as the threshold values to categorize samples into low or high TPTE expression.

### Cell culture

The MCF-7 (human breast cancer) and PC-3 (human PCa) cell lines procured from the National Cell Bank of Pasteur Institute, Iran, were cultured in Dulbecco’s modified Eagle’s medium (DMEM) supplemented with penicillin and streptomycin (Gibco, UK) and 10% fetal bovine serum (FBS) (Gibco, UK). The culturing process took place at 37 °C in a humidified atmosphere containing 5% CO_2_ and 95% air.

### Flow cytometry

To assess the percentage of cells expressing TPTE protein, indirect flow cytometry was performed on MCF-7 and PC-3 cells using an anti-TPTE antibody (LSBio, USA). The test involved isolating adherent cells using 0.25% trypsin–EDTA (Gibco, UK) at 37 °C for 1 min. The cells were washed with FACS buffer (3% BSA/PBS), followed by incubation with an anti-TPTE antibody at 10 µg/ml on ice for 30 min. After two additional washes with FACS buffer, a fluorescently labeled secondary antibody (PE-conjugated goat anti-rabbit IgG; Biolegend, USA) was added and incubated on ice for 30 min. Subsequently, the cells underwent analysis for TPTE expression using FlowJo software (Bagherian et al. 2022). Two control groups were implemented: one with Ig G fraction as primary antibody (isotype control), and the other with PBS as the primary antibody instead of the anti-TPTE antibody (unstained). These controls served the purpose of distinguishing between non-specific background fluorescence and authentic TPTE protein expression in the cells.

### Cell viability

The cell viability was assessed using a 3-(4,5-dimethylthiazol-2-yl)-2,5-diphenyltetrazolium bromide (MTT) assay kit from Bio Idea Co., Iran. MCF-7 and PC-3 cells were seeded in 96-well plates at a density of 5 × 10^3^ cells per well in 200 μl of DMEM and incubated overnight at 37 °C. Subsequently, the cells were treated with varying concentrations of the anti-TPTE-p2 antibody (2.5, 5, and 10 µg/ml) or PBS buffer (control) in 200 μl of fresh medium for 24 or 48 h. Following treatment, MTT solution (20 μl) was added to all wells, and incubation continued for an additional 4 h. The culture medium was then removed, and each well was exposed to dimethyl sulfoxide (DMSO) (100 μl) to dissolve the frozen crystals. The absorbance of the samples was measured at 570 nm with a reference filter of 630 nm. This experimental procedure was repeated three times, and cell viability was calculated using the formula: (mean OD sample/ mean OD control) × 100 (Kamiloglu et al. 2020).

### Statistical analysis

Graphs were generated using Prism 8.3.0 software, and data analysis was conducted with SPSS 26. A two-way analysis of variance (ANOVA) was employed for comparing ELISA data. The correlation between clinicopathological parameters and TPTE expression underwent analysis through Pearson’s χ^2^ and Pearson’s R tests. Group comparisons were executed using Kruskal–Wallis and Mann–Whitney U tests. Survival analysis utilized the Kaplan–Meier method, and resulting curves for each group were compared using the log-rank test. Flow cytometry and MTT assay experiments were performed in triplicates. All data were presented as mean ± standard deviation (SD), and statistical significance was determined by a P-value < 0.05.

## Results

### Polyclonal antibody against TPTE sequences

**Fig. 1:**
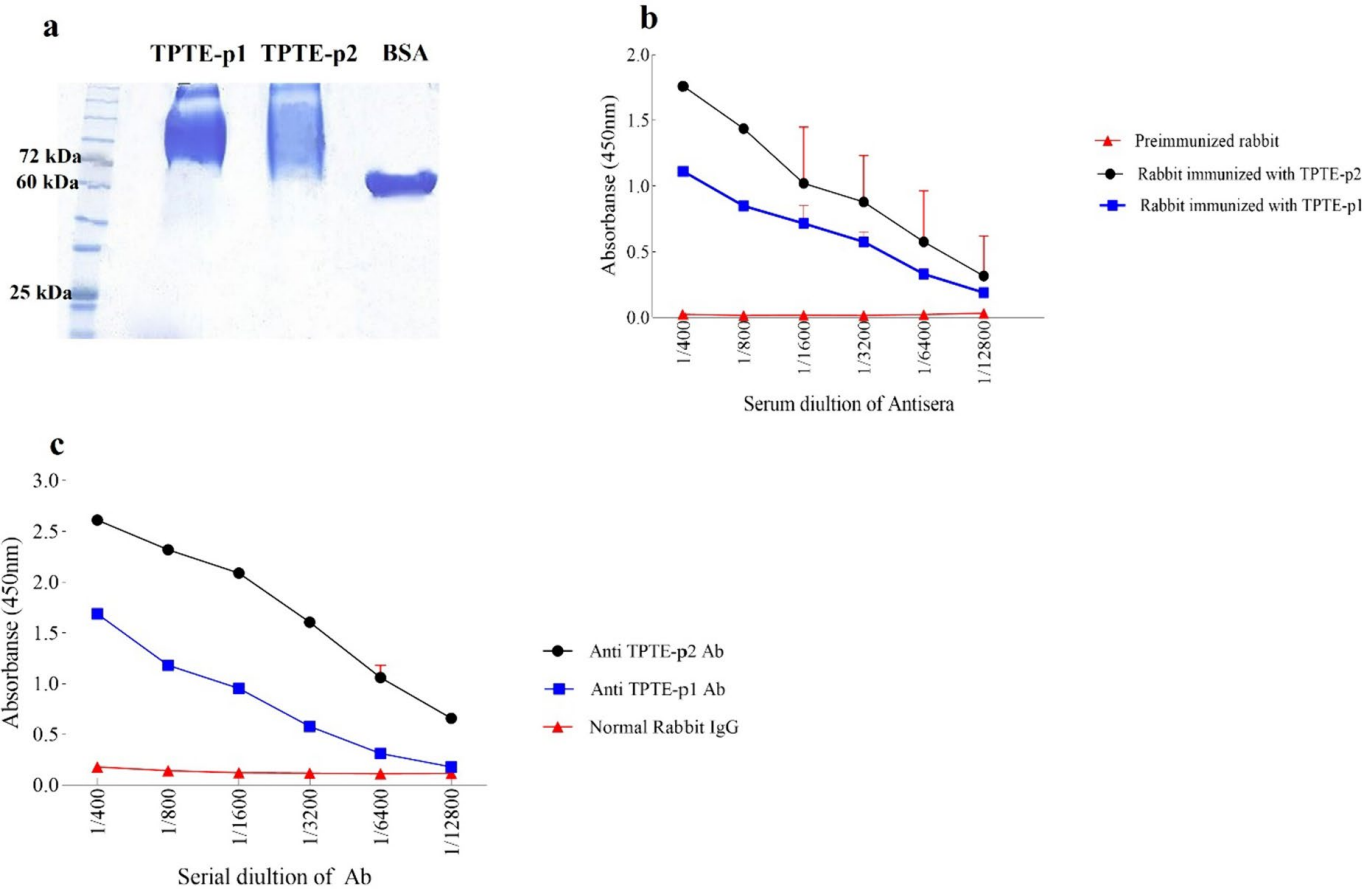
The conjugated TPTE-p1 and p2 peptides underwent analysis using gel electrophoresis and indirect ELISA for antibody production and the production of antibodies against them were assayed using gel electrophoresis and indirect ELISA, respectively. **a** Conjugation with BSA resulted in smear bands heavier than BSA weight (66 kDa). **b** After the first booster injection, titration of anti-TPTE-p1 and p2 antisera showed consistently high titers at 1/6400 (P < 0.001) and 1/12800 (P < 0.001) dilutions, respectively. after the first booster injection. **c** Following purification using affinity chromatography, titration of anti-TPTE-p1 and p2 antibodies indicated significantly elevated titers at 1/6400 (P < 0.01) and 1/12800 (P < 0.001) dilutions, respectively. Statistical analysis by Two-way Anova supported the robustness of the results

The SDS-PAGE technique was employed to investigate the conjugation process of TPTE peptides with BSA prior to rabbit immunization. In Fig. 1a, the binding of peptides to BSA resulted in the formation of heavier molecules, positioned above the molecular weight of BSA (66 kDa) on the electrophoresis gel. Subsequently, polyclonal antibodies were generated in rabbits in response to immunogenic BSA-conjugated TPTE-p1 (peptide fragment 421–436) and TPTE-p2 (peptide fragment 514–531). Two rabbit antisera, anti-TPTE-p1 and anti-TPTE-p2, exhibited immunoreactivity in an indirect ELISA assay, contrasting with the non-reactive pre-immunized serum from the same rabbit (control group). ELISA results demonstrated significantly elevated antibody levels compared to the control (Fig. 1b). Titers of TPTE-p1 and TPTE-p2 antisera were consistently high at 1/6400 (P < 0.001) and 1/12800 (P < 0.001) dilutions, respectively. Affinity chromatography was employed for antibody purification, with IgG from a pre-immunization rabbit serving as a negative control. The purified anti-TPTE-p1 and anti-TPTE-p2 antibodies exhibited significantly elevated titers at dilutions of 1/6400 (P < 0.01) and 1/12800 (P < 0.001), respectively (Fig. 1c).

### Study population

The average age of the study population was 67 years (ranging from 46 to 87 years). PCa Patients, as well as those with BPH, had a mean age of 67 years (ranging from 46 to 97 years and 53 to 85 years, respectively). PSA levels were categorized as < 4, 4–10, and < 10 ng/ml (with a range of 0.2–1275 and a mean of 43.22 ng/ml). Among the 85 cases with available PSA data, 13% (11) had a PSA < 4 ng/ml, 40% (34) PSA of 4–10 ng/ml, and 47% (40) PSA < 10 ng/ml. Additionally, in 102 cases of PCa, 17.6% (18) exhibited a Gleason score of 6, 24.5% (25) score of 7, 14.7% (15) a score of 8, 27.4% (28) a score of 9, and 14.7% (15) a score of 10 (Table 1).

**Table 1:** Association between TPTE expression (intensity, percentage of positive cells and H-score) and clinicopathological parameters of PCa (P value; Pearson χ^2^)

Patients and tumor characteristics	No (%)	Cytoplasmic expression of TPTE *(P value)*			Membrane expression of TPTE *(P value)*		
		Intensity	Percentage of positive cells	H-score cut-off = 188	Intensity	Percentage of positive cells	H-score cut off = 134.88
Age (years)		0.89	0.77	0.57	**0.03**	0.12	0.1
< 66	28 (27.45)						
> 66	73 (71.56)						
Unknown	1 (0.98)						
Gleason scores		0.25	0.1	**0.03**	0.92	0.95	0.43
Gleason score 6	18 (17.6)						
Gleason score 7	25 (24.5)						
Gleason score 8	15 (14.7)						
Gleason score 9	28 (27.4)						
Gleason score 10	16 (15.68)						
PSA (ng/ml)		**0.06**	0.13	**0.001**	**0.04**	**0.03**	**0.01**
< 4	11 (10.9)						
4–10	34 (33.3)						
> 10	40 (39.2)						
Unknown	17 (16.6)						
Perineural Invasion (PNI)		0.9	0.06	0.51	0.2	0.26	**0.04**
Present	46 (45)						
Absent	56 (55)						
Extra prostatic Extension (EPE)		0.5	0.51	0.41	0.2	0.63	0.73
Present	18 (17.6)						
Absent	84 (82.4)						

Values in bold are significant

*No* number; *H- score* Histochemical score

### Expression of TPTE in PCa and BPH tissues

To investigate the expression of TPTE as a specific tumor marker in PCa tissues, we conducted immunohistochemical analysis. The evaluation of TPTE expression levels utilized the H-score. It is worth noting that the anti-TPTE-p1 antibody did not yield a suitable staining pattern in immunohistochemistry, leading to its exclusion from the experiment. Consequently, only the anti-TPTE-p2 antibody was employed for the remaining steps of the study.

**Fig. 2:**
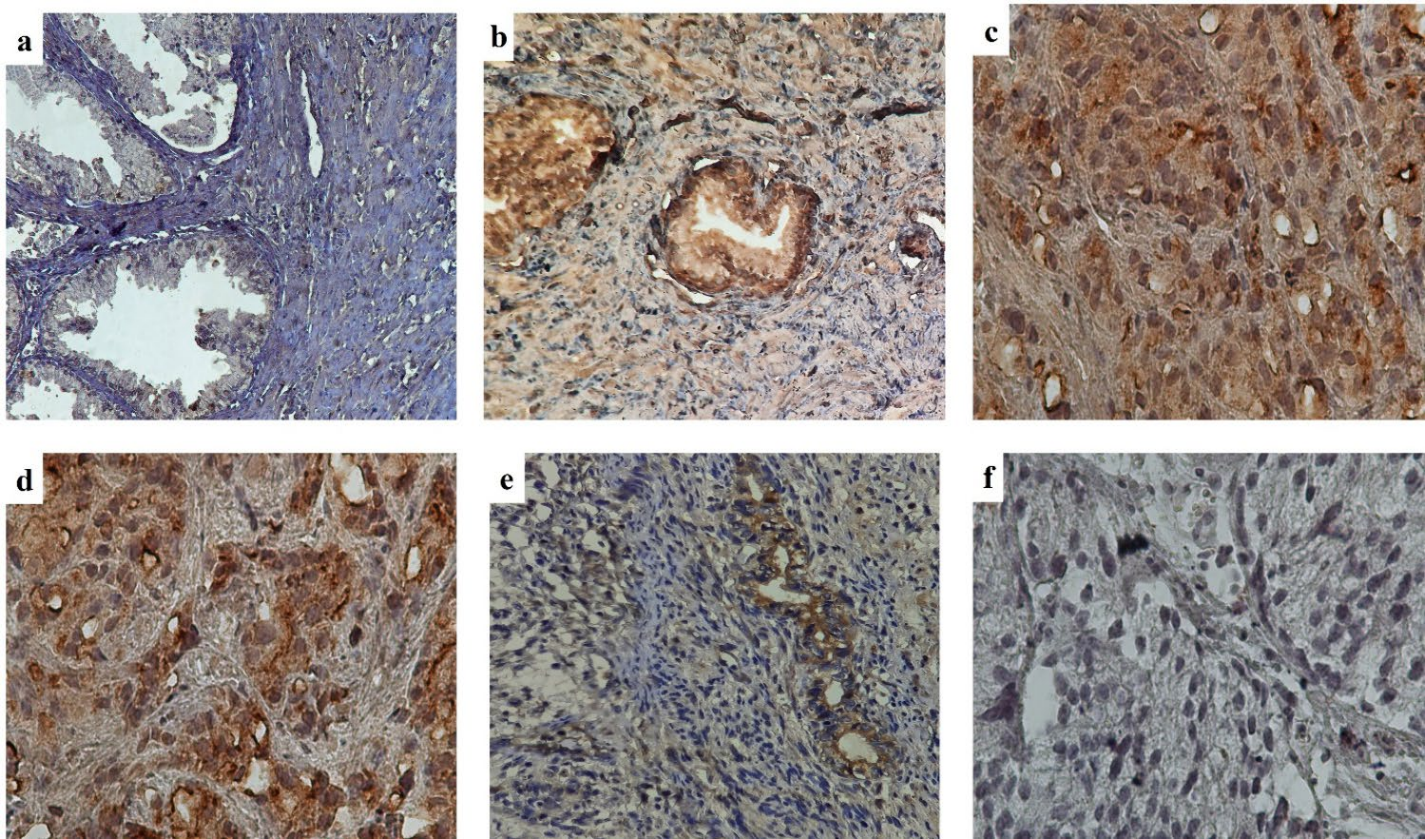
Immunohistochemical analysis of staining pattern of TPTE expression. **a** Weak staining in BPH, **b** moderate staining in PCa tissue with Gleason score 6, **c** strong staining in PCa tissue with Gleason score 8, **d** TPTE staining in PCa tissue by commercial antibody (LSBio, USA) for a comprison to the Ab in the study, **e** TPTE expression in testis tissue as positive control, and **f** negative isotype control in PCa tissue. All images are prepared at original magnification × 400

**Fig. 3:**
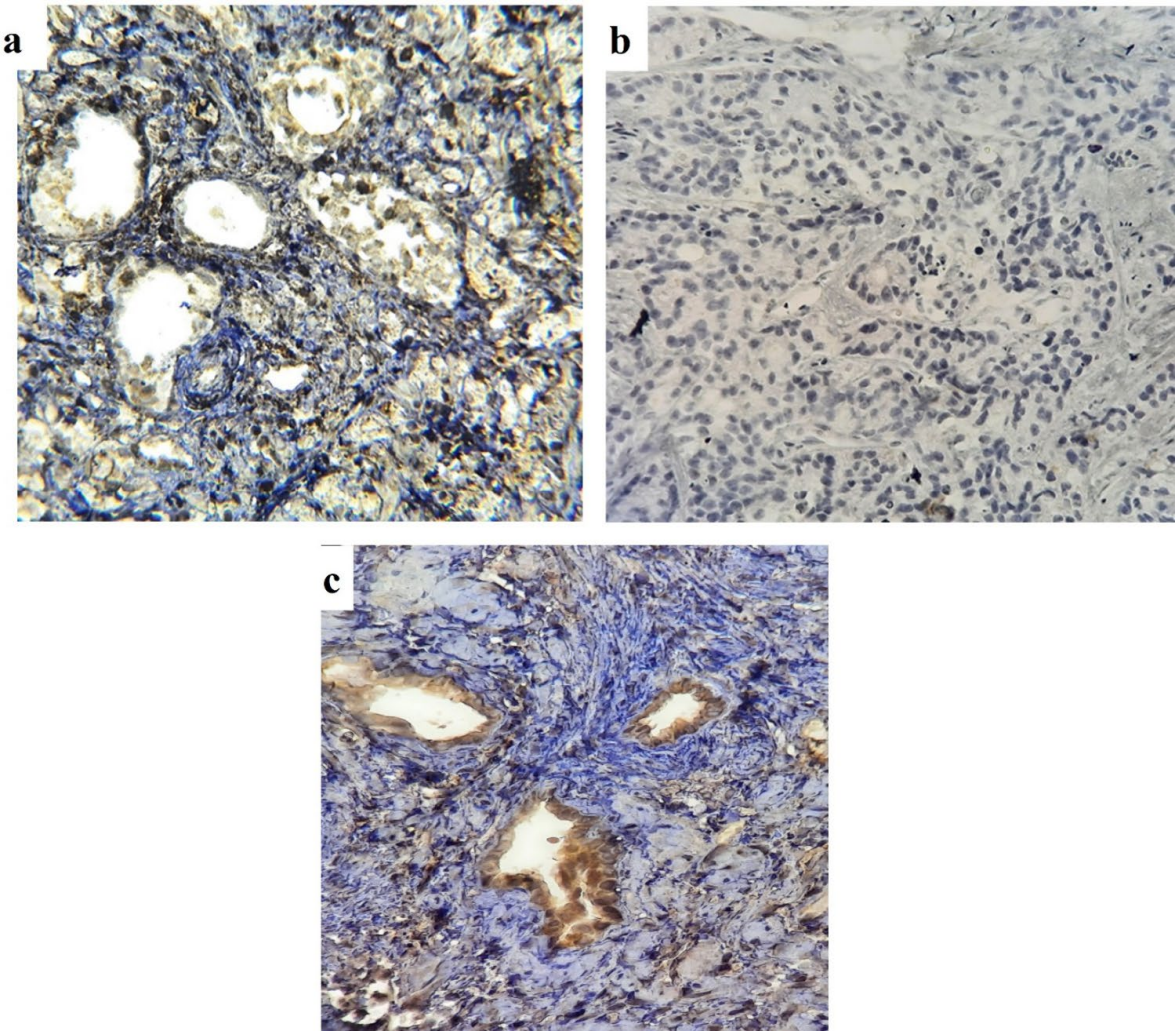
Assessing anti-TPTE-p2 antibody binding inhibition through pre-incubation with TPTE-p2 peptide before exposing it to prostate tissue. Three conditions were examined: **a** pre-incubation with 10 μg/ml of TPTE-p2, **b** pre-incubation with 20 μg/ml of TPTE-p2, and **c** no pre-incubation. The results showed weak staining in (**a**) no staining in (**b**) and staining of prostate tissue in (**c**) when using the anti-TPTE-p2 antibody without TPTE-p2 pre-incubation. This observation at an original magnification of × 400 indicates the specificity of the anti-TPTE-p2 antibody for the regions of interest

In the examination of TPTE expression in both PCa and BPH tissues, the anti-TPTE-p2 antibody predominantly stained the membrane (79% of tissues) and cytoplasm (100% of tissues) of epithelial cells in PCa as well as in in BPH samples, displaying varying intensities. Additionally, a nuclear staining pattern for TPTE was detected in 24% of the samples. Anti-TPTE-p2 antibody mainly stained the membrane (79% of tissues) and cytoplasm (100% of tissues) of epithelial cells of PCa and BPH samples with different intensities (Fig. 2). To confirm the specificity of the anti-TPTE-p2 antibody, a peptide blocking assay was conducted, revealing a decrease in tissue staining intensity with an increase in peptide concentration (Fig. 3). Among the 102 PCa samples stained for TPTE, different intensities of expressions were found in cytoplasmic membrane, and nucleus. The level of expressions for cytoplasmic membrane as weak, moderate, and strong intensities were observed in 9.8% (10), 49% (50), and 36.2% (37) of cases respectively. Membrane expression was detected in 10% (7), 27% (18), %37 (25), and 25% (17) of the cases with negative, weak, moderate, and strong intensities, respectively. Of 38 PCa samples a nuclear pattern of TPTE expression, as weak, moderate, and strong intensities were observed in 36.8% (14), 31.6% (12), and 28.9% (11) of cases, respectively. Similarly, BPH samples exhibited varied staining intensities for cytoplasmic and membrane expression, with a rare occurrence of nuclear staining. Of 48 BPH samples, weak staining for cytoplasmic expression was observed in 79.2% (38), whereas moderate and strong staining were detected in 14.9% (7) and 4.2% (2) of cases, respectively. Membrane expression of TPTE was absent in 30% (9 cases), but weak expression exhibited in 43.3% (13 cases), moderate in 23.3% (7 cases), and strong in 3.3% (1case). Only four BPH samples showed nuclear staining: two with weak and two with moderate staining.

**Fig. 4:**
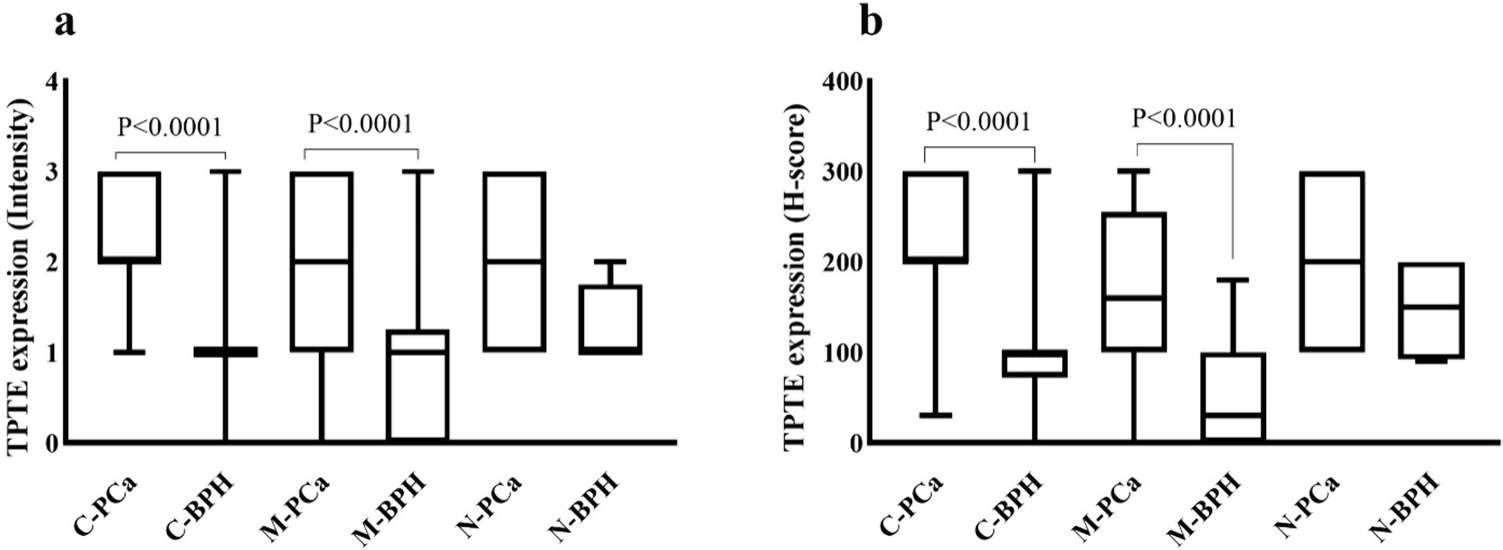
Immunohistochemical analysis of TPTE expression in PCa and BPH groups. **a** Comparative analysis of TPTE expression between PCa and BPH samples based on staining intensity and **b** based on H-score. The Mann–Whitney U test was performed, and only the statistically significant P-values are displayed in the figure. *C-PCa*, *M-PCa*, and *N-PCa* stand for cytoplasmic, membrane, and nuclear expression in prostate cancer, respectively. *C-BPH*, *M-BPH*, and *N-BPH* stand for cytoplasmic, membrane, and nuclear expression in benign prostatic hyperplasia, respectively

The mean intensity of cytoplasmic, membrane, and nuclear TPTE expression was higher among PCa samples (mean ± SD: 2.27 ± 0.64, 1.88 ± 0.94, and 1.97 ± 0.84, respectively) compared to BPH (1.17 ± 0.6, 0.86 ± 0.86, and 1.5 ± 0.5, respectively). The mean H-score for cytoplasmic, membrane, and nuclear expression was (219.5 ± 62.6, 173.8 ± 89.3, and 196.3 ± 82.3) in PCa cases and (98.9 ± 59.6, 58.5 ± 40, and 147.5 ± 60) in the BPH group, respectively. Statistical analysis using the Mann–Whitney U test indicated significant differences in H-score and staining intensity between PCa cases and the BPH group for both cytoplasmic (*P* < 0.0001) and membrane TPTE expression (*P* < 0.0001). There was no significant difference in the nuclear staining pattern between PCa and BPH in terms of H-score (P = 0.7) and staining intensity (P = 0.9) (Fig. 4a and b).

### Association of TPTE expression in terms of H-score with clinicopathological parameters

**Fig. 5:**
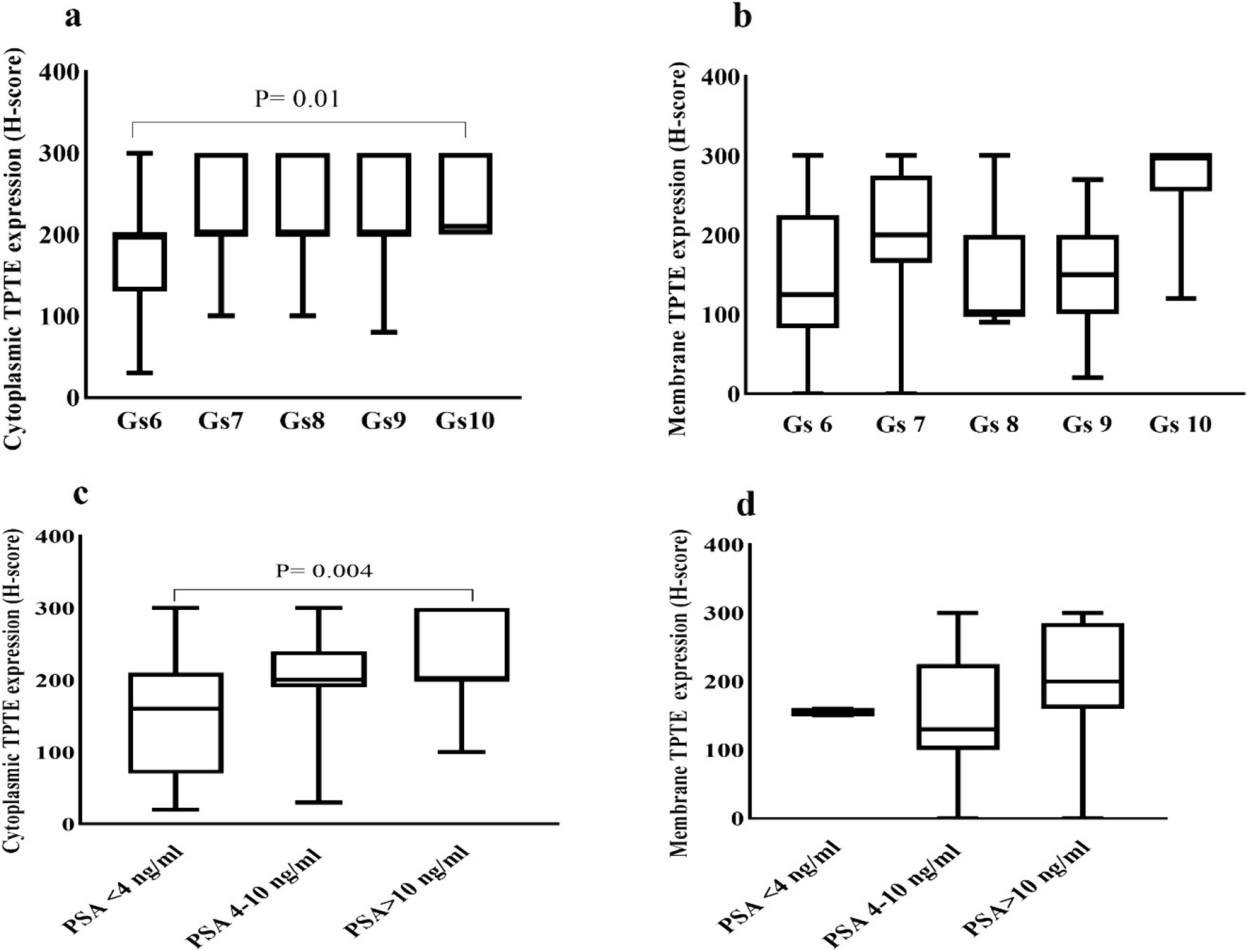
Immunohistochemical analysis of TPTE expression in terms of H-score in PCa. **a** Correlation between Gleason scores and cytoplasmic and **b** membrane expression of TPTE using the Kruskal–Wallis test. **c** Association of PSA level and cytoplasmic and **d** membrane expression of TPTE using the Kruskal–Wallis test. Only the significant *P*-values are indicated in the figures. *Gs* Gleason score, *H- score* Histochemical score, *PSA* Prostate-specific antigen

Further analysis involved exploring the correlation of TPTE expression (in terms of H-score) with clinicopathological parameters. Significant positive correlations were found between cytoplasmic TPTE expression and the Gleason score, as well as PSA levels. The Kruskal–Wallis test revealed noteworthy variations in cytoplasmic H-score among different Gleason scores. To analyze the association of TPTE expression in terms of H-score with clinicopathological parameters, Pearson’s χ^2^ analysis was conducted. It is noted that the nuclear expression was excluded from statistical analysis, due to a small percentage of cases and analysis was mainly performed on cytoplasmic and membrane TPTE expression. The results revealed a significant positive correlation between the cytoplasmic expression of TPTE and the Gleason score (P = 0.03, Fig. 5a) (Table 1). However no significant correlation was found between membrane TPTE expression and Gleason score (P = 0.43, Fig. 5b). The Kruskal–Wallis analysis indicated a difference between Gleason scores 6 and 10 in cytoplasmic H-score (P = 0.01) (Fig. 5a).

Pearson’s χ^2^ analysis also demonstrated a statistically significant relationship between PSA levels in both cytoplasmic and membrane H-scores (P = 0.001 and P = 0.01, respectively) (Table 1). Using the Kruskal–Wallis test, a significant distinction was detected between the PSA in 4 ng/ml group and 10 ng/ml group (P = 0.004) in regard to cytoplasmic TPTE expression (Fig. 5c); however, this analysis did not show a significant relationship between the membrane H-score and PSA groups (P = 0.4) (Fig. 5d). Pearsons χ^2^ analysis indicated a notable difference between perineural invasion (PNI) and Membrane H-score (P = 0.04); however, this distinction did not reach significance in terms of cytoplasmic expression (P = 0.51). Additionally, statistical analysis did not show significant correlations between extraprostatic extension (EPE) and both cytoplasmic and membrane H-scores (P = 0.41 and P = 0.73, respectively) (Table 1).

### Correlation of TPTE expression and PCa prognosis based on H-score

**Fig. 6:**
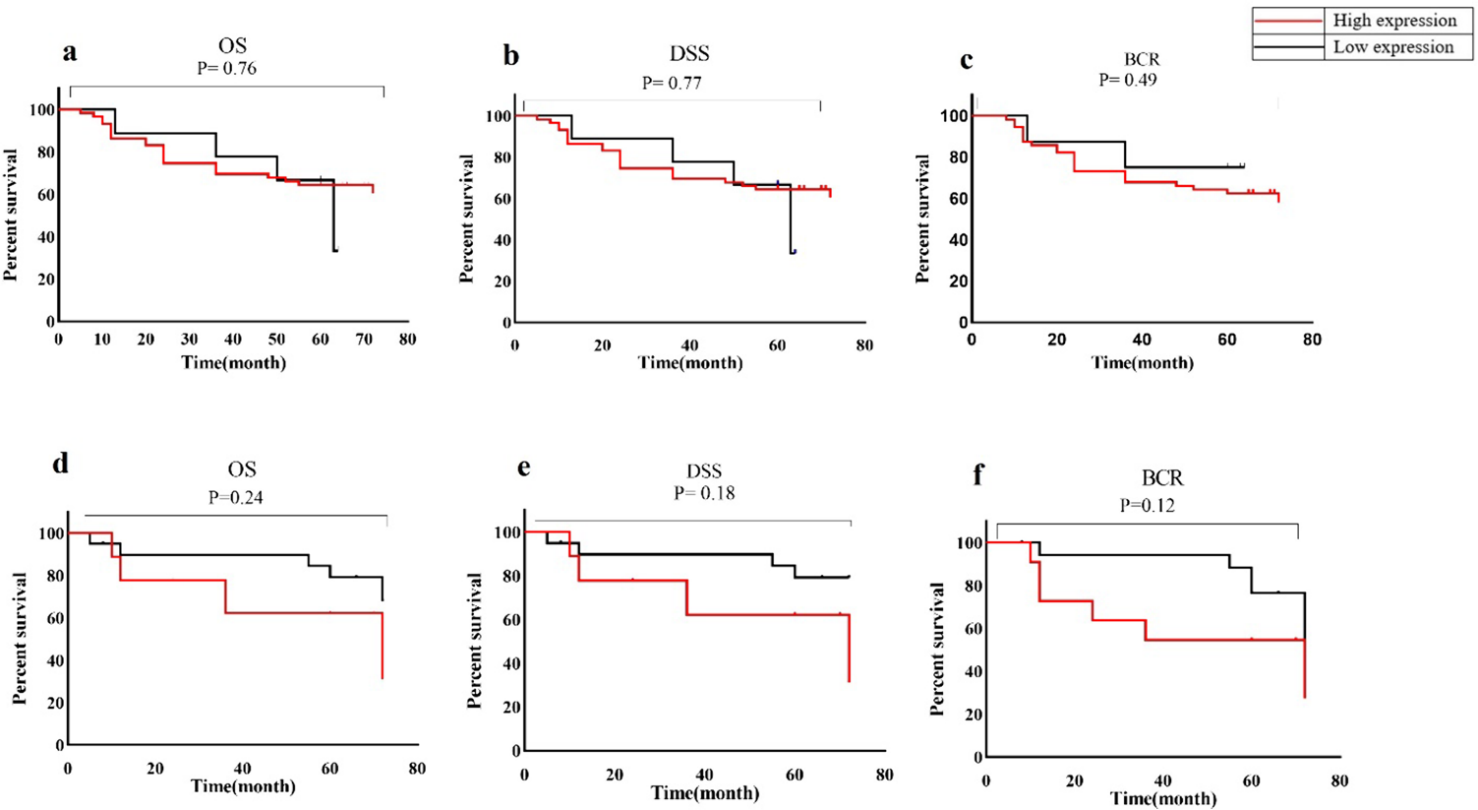
Survival analysis for TPTE expression in PCa patients. **a** Kaplan–Meier curves of cytoplasmic TPTE expression for OS, **b** DSS, and **c** BCR. **d** Kaplan–Meier curves of membrane TPTE expression for OS, **e** DSS, and **f** BCR. Expression of TPTE was grouped into low and high expression levels based on H-score cut-off. *OS* overall survival*, DSS* disease-specific survival,* BCR* biochemical recurrence

The study utilized Kaplan–Meier analysis, specifically the log-rank test, to explore the correlation between TPTE expression and DSS, OS, and BCR. Patients were categorized into low and high TPTE expression groups using cytoplasmic and membrane mean H-scores. Due to its infrequent occurrence (24%) in the examined samples, the nuclear expression pattern was excluded from survival studies. Data for DSS, OS, and BCR were collected from primary treatment or surgery until the end of 2022. The mean postoperative follow-up period was 64.9 ± 25.84 months, with a median of 60 months and a range of 5–72 months. Key patient characteristics for the survival analysis are summarized in Table 2. In patients with low or high TPTE cytoplasmic expression, the mean BCR durations were 52 ± 22.94 and 70.69 ± 22.58 months, respectively. Similarly, the mean OS durations for low and high TPTE cytoplasmic expression were 46.22 ± 21.69 and 63.63 ± 19.59 months, respectively. The average DSS rates for individuals exhibiting low and high cytoplasmic TPTE expression were 55.2 ± 13.71 and 69 ± 21.87 months, respectively. Figure 6A illustrates a decreased survival among patients with elevated cytoplasmic TPTE levels. Although survival rates tended to decrease with high cytoplasmic and membrane TPTE expression, no significant correlations were found with DSS, OS, or BCR (P = 0.76, P = 0.77, and P = 0.49, respectively, Fig. 6). In cases of low membrane TPTE expression, the mean BCR was 52 ± 22.94 months, whereas high expression resulted in 70.69 ± 22.58 months. Similarly, individuals with low TPTE membrane expression had a mean OS of 49.82 ± 22.69 months, compared to 59.16 ± 20 months in those with high expression. The mean DSS rates for low and high membrane TPTE expression were 55.2 ± 13.71 and 69 ± 21.87 months, respectively. Similar to the cytoplasmic pattern, high membrane TPTE expression correlated with lower survival rates; however, statistical significance was not achieved in the differences between membrane TPTE expression and OS, DSS, or BCR (P = 0.24, P = 0.18, and P = 0.12, respectively, Fig. 6).

**Table 2:** The main Characteristics of PCa patients included for survival analysis

Number of patients (N)	69
Duration of follow up time (mean, SD)	64.9 ± 25.84
Duration of follow up time (median)	60 months
Number of BCR (%)	25 (36.3%)
Number of metastasis patients (%)	4 (5.7%)
Number of alive patients without any complication (%)	43 (62.3%)
Number of deaths related to PCa (%)	22 (31.8%)

*OS* overall survival; *DSS* disease-specific survival; *BCR* biochemical recurrence

### TPTE expression in cancer cell lines

**Fig. 7:**
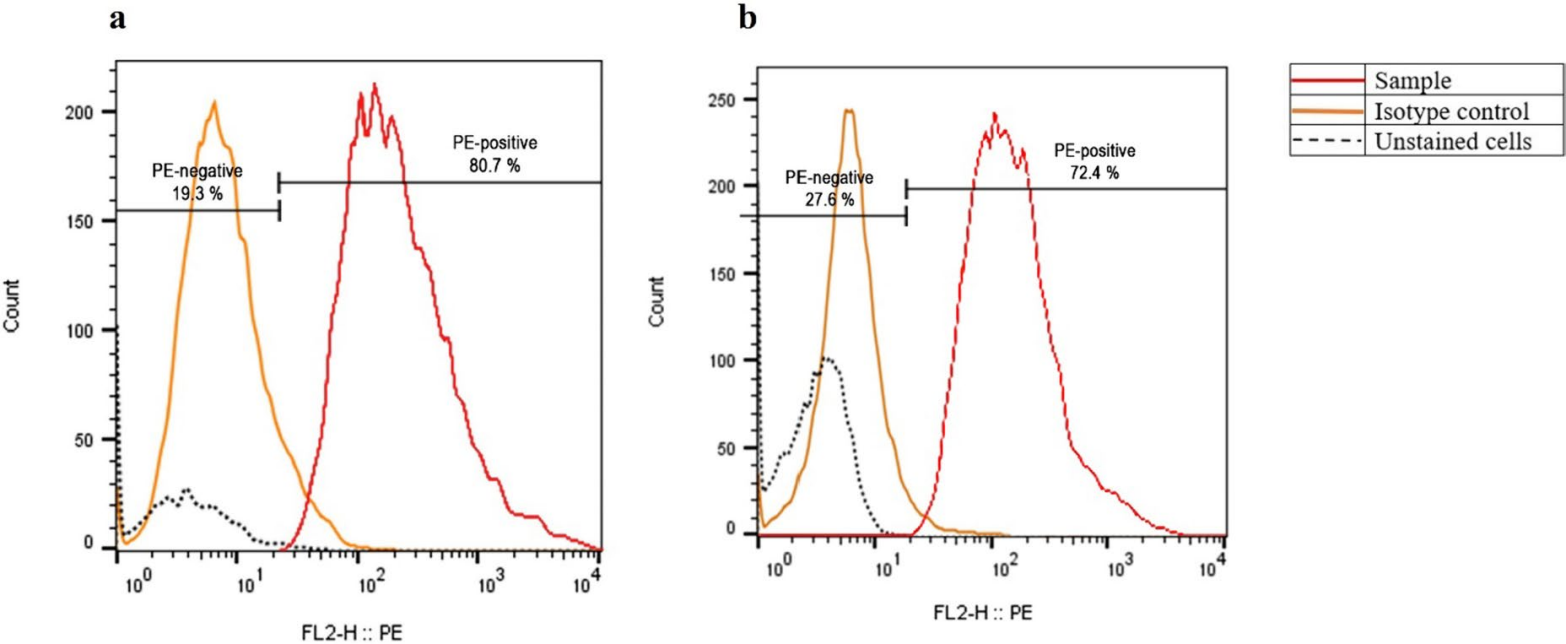
Conducting flow cytometric analysis, TPTE expression examined in MCF-7 and PC-3 cells. The surface expression of TPTE was investigated using the anti-TPTE antibody at a concentration of 10 µg/ml (LSBio, USA) in MCF-7 (**a**) and PC-3 cells (**b**). To ensure specificity, control groups included cells exposed to PBS (unstained) or IgG fraction (isotype control) as the primary antibody

The expression of TPTE in cancer cell lines was investigated through flow cytometric analysis in three specific cell lines (MCF-7 and PC-3). The anti-TPTE antibody (10 µg/ml) was utilized for this analysis. The test results were compared with control groups, the cells that were exposed to PBS (unstained) or Ig G fraction (isotype control) as primary antibody. The findings revealed that the surface expression of TPTE was observed in 80% ± 0.9 of MCF-7 cells and 72.4% ± 0.8 of PC-3 cells (Fig. 7).

### Anti -TPTE-p2 effects on cell viability of cancer cell lines

**Fig. 8:**
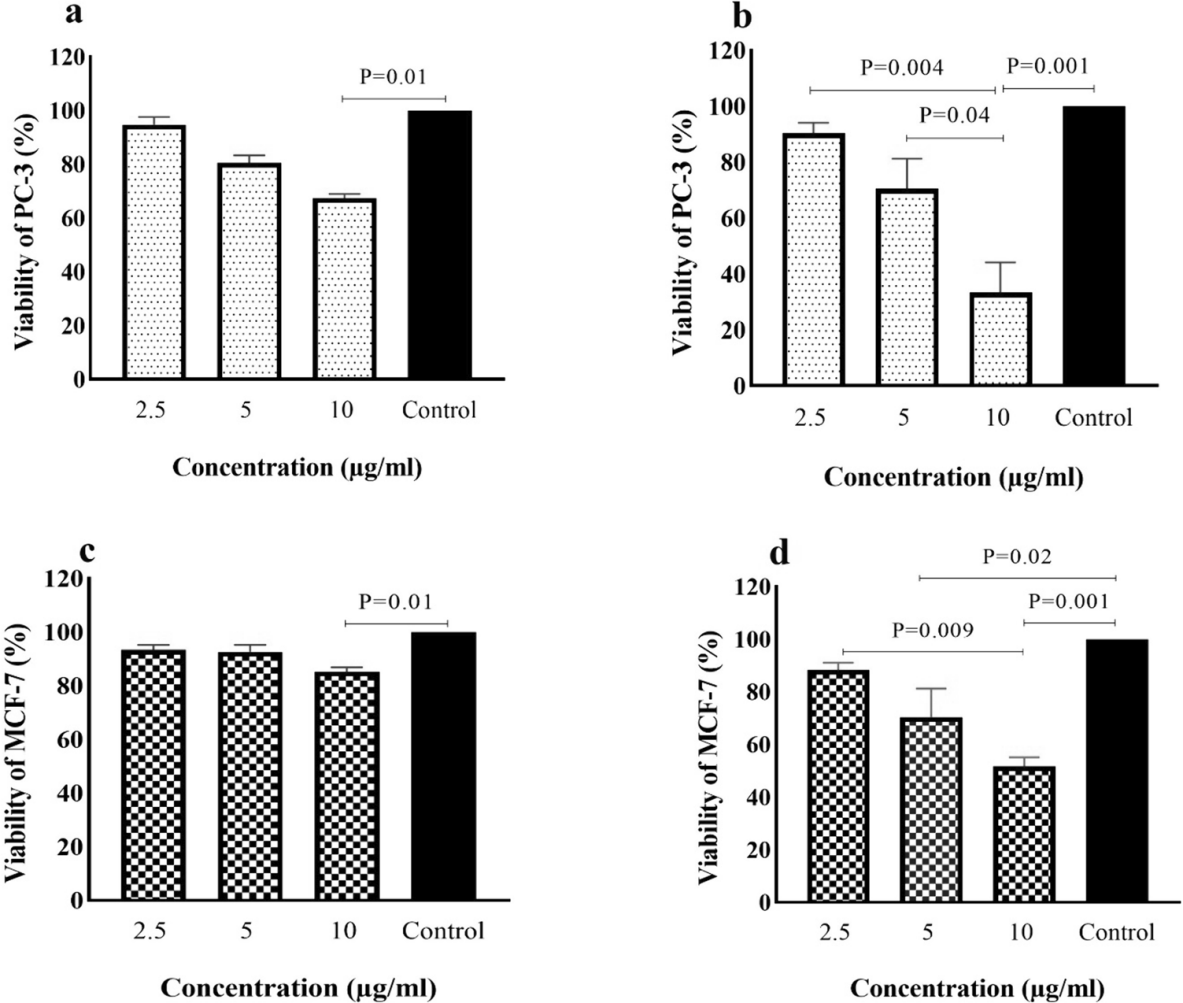
Exploring the impact of the anti-TPTE-p2 antibody on cell viability. **a** Assessment of PC-3 cell viability 24 h post-administration and **b** at 48 h. Similarly, **c** Evaluation of MCF-7 cell viability at 24 h and **d** at 48 h post anti-TPTE-p2 antibody administration. In comparison to the control group treated with PBS, all cells exhibited reduction of viability. However, noticeable reductions exhibited in the figures. Statistical analysis was conducted using the Kruskal–Wallis test

The potential cytotoxic impact of the anti-TPTE-p2 antibody on cancer cell lines was assessed using the MTT assay. MCF-7and PC-3 cells were exposed to varying concentrations of the antibody (2.5, 5, 10, and 15 μg/ml) for either 24 or 48 h. Results showed a significant inhibition in the proliferation of both PC-3 and MCF-7 cells after 24 h of exposure to the anti-TPTE-p2 antibody compared to the control group receiving PBS (P < 0.001 and P = 0.001, respectively). Furthermore, at a concentration of 10 μg/ml, cell viability was significantly reduced in both PC-3 and MCF-7 cell groups compared to the control (P = 0.01) (Fig. 8a and c). After 48 h, a noteworthy increase in cell mortality was observed in both PC-3 and MCF-7 cells compared to the control group (P = 0.001 and P = 0.009, respectively) (Fig. 8b and d). Additionally, significant reductions in cell viability were noted in the PC-3 group at 10 μg/ml (P = 0.001) and the MCF-7 group at 5 (P = 0.02) and 10 μg/ml (P = 0.001) compared to the control. The maximum mortality rate, around 60%, was observed in PC-3 cells treated with 10 μg/ml, significantly higher than the control group (P < 0.001). These results indicate a positive correlation between cell death rate and both the concentration and duration of anti-TPTE-p2 antibody treatment (Fig. 8).

## Discussion

CTAs hold promise as targeted molecules for personalized immunotherapy and as prognostic or diagnostic biomarkers for PCa. Among these antigens, the TPTE family are particularly attractive targets due to their robust immunogenic properties and limited expression in normal tissues. Nonetheless, the role of TPTE is still poorly understood where CTAs are emerging as potential tumor markers for various malignant tumors, showcasing the continuous exploration of novel diagnostic and therapeutic avenues. TPTE is implicated in cell signaling modulation through interactions with membrane-bound molecules, exerting its influence via de-phosphorylation processes (Atanackovic et al. 2006). This multifaceted role extends to the association between elevated TPTE expression and enhanced survival outcomes, a phenomenon observed across various cancer types. The cancer/testis antigen TPTE is aberrantly expressed in various tumors, including lung cancer, implicating its involvement in the immune responses against cancer cells (Dong et al. 2003; Bansal et al. 2015). TPTE is considered a member of the PTENs family, which shares lineage with the well-known PTEN. Situated on chromosome 10, PTEN plays a pivotal role in regulating the cell cycle and proliferation. Importantly, the common association of PTEN loss with tumor development underscores its significance in the tumorigenesis process (Gao et al. 2021).

Understanding the intricate TPTE signaling pathways involved in PCa provides potential diagnostic tumor marker to intervene in disease progression. Hence, in our investigation, we identified specific extracellular regions of TPTE that demonstrate the highest effectiveness in initiating an immune response in B cells, as determined by IEDB algorithms. Peptide fragments covering amino acid sequences 421–436 and 514–531 were pinpointed as crucial regions. These specific segments displayed robust efficacy in activating B cell immune responses. ELISA tests validated the significant immunogenicity of both TPTE-p1 and p2 by generating elevated antibody levels.

In this research, a novel discovery emerged as we identified a correlation between the cytoplasmic expression level of TPTE and crucial prognostic indicators in PCa, including the Gleason score and PSA level. This finding shed light on the potential significance of TPTE in understanding and predicting the prognosis of PCa patients. Aligned with prior investigations (Adepiti and Odunsi 2022) our study reveals a significant increase of TPTE expression in PCa samples compared to BPH samples. The pivotal discovery of our research lies in the substantial correlation we identified—higher TPTE expression is significantly associated with escalated disease severity, as evidenced by elevated Gleason scores. Furthermore, a significant association was observed between the severity of the disease and PSA level. Elevated PSA levels were consistently linked to heightened protein expression and H-score. We also found a significant positive correlation between TPTE membrane expression and the pathological index of PNI, suggesting that this protein may play a role in the progression of PCa. Adepiti et al. conducted a study employing RT-PCR to assess TPTE expression in ovarian tumors. Similar to our findings, they identified TPTE expression in a significant proportion of tumors (45.1%). However, their study did not establish significant correlations between TPTE expression and variables such as patient age, tumor stage, grade, histology, or response to therapy (Adepiti and Odunsi 2022). Nonetheless, these findings suggest that TPTE could be a potential marker for tracking the progression of PCa.

In a computational investigation, researchers explored the genes associated with clinical features and prognosis in oral squamous cell carcinoma (OSCC) using RNA-sequencing data. Through comprehensive analyses, they identified 17 Gene Ontology terms and 4 KEGG pathways using functional enrichment and protein–protein interaction network analysis. Notably, six specific genes—DCAF4L2, OPRPN, R3HDML, TPTE, ACTL8, and PCDHA11—emerged as having a substantial impact on patient survival. Remarkably, their results underscore a positive correlation between the presence of TPTE and an enhanced likelihood of increased patient survival (Wu et al. 2019). Additionally, in the Adepiti et al. study, a pattern emerged suggesting a connection between TPTE expression and slightly prolonged progression-free survival (PFS) and OS. Notably, it is crucial to emphasize that this correlation did not achieve statistical significance (Adepiti and Odunsi 2022). In our research, we observed that patients with elevated H-scores of TPTE expression exhibited diminished survival rates; however, this discrepancy lacked statistical significance. The PTEN gene holds a pivotal function in overseeing cell cycle regulation and the proliferation of cells. Importantly, the noteworthy association between the diminished expression of PTEN and the onset of tumor development underscores its substantial role in the intricate process of tumorigenesis (Gao et al. 2021). Consequently, there is a belief that TPTE, akin to PTEN, could exert a noteworthy influence on the progression of cancer. To achieve a thorough comprehension of the specific role played by the TPTE protein, additional in-depth studies are deemed necessary.

To check cell viability, in addition to prostate cell line, we also used breast cell line to study the different effect of the anti-TPTE-p2 antibody on these two cell lines. We conducted experiments exposing PC-3 and MCF-7 cells to the anti-TPTE-p2 antibody to demonstrate its efficacy against cancer. The results revealed that varied doses of the anti-TPTE-p2 antibody effectively restrained the proliferation of both PC-3 and MCF-7 cell lines. Notably, the inhibitory effect demonstrated a direct correlation with the antibody dosage, indicating more pronounced suppression at higher doses. These findings underscore the potential of the anti-TPTE-p2 antibody as a promising inhibitor for curtailing the growth of PCa and breast cancer cells, offering valuable insights for innovative treatment development. Additionally, Sahin and collaborators (2020) conducted phase I trials testing the melanoma liposomal RNA vaccine (BNT111) against four non-mutated antigens, including TPTE, Tyrosinase, melanoma-associated antigen A3 (MAGE-A3), and New York Esophageal Squamous Cell Carcinoma 1 (NY-ESO-1). The study revealed that the combination of these antigens elicited a robust immune T-cell response, with heightened CD4 + T-cell responses against MAGE-A3, TPTE, and NY-ESO-1, and CD8 + T-cell responses against NY-ESO-1 and MAGE-A3. Moreover, the vaccine, either independently or combined with checkpoint inhibitors, successfully induced persistent immune responses in individuals diagnosed with advanced stage 3 and 4 melanoma cancer (Sahin et al. 2020). These cumulative findings underscore the significance of TPTE as a pivotal factor in cancer diagnosis, prognosis, and therapy, laying a robust groundwork for ongoing research and the advancement of groundbreaking management of PCa patients.

## Conclusion

In summary, this comprehensive study provides insights into the expression patterns of TPTE in PCa and BPH tissues, highlighting its potential associations with clinicopathological parameters and its impact on cancer cell viability. Elevated TPTE levels may correlate with the onset of PCa, hence it can be used as a potential protein biomarker to assess the disease process. Additional research is crucial to comprehensively understand and validate the specific functional mechanisms of TPTE in cancer development across diverse cancer types.

## Data Availability

The data presented in this study are available on request from the corresponding author. The data are not publicly available due to a privacy issue from the patients. No datasets were generated or analyzed during the current study.
